# Toward a Functional Cure for Hepatitis B: The Rationale and Challenges for Therapeutic Targeting of the B Cell Immune Response

**DOI:** 10.3389/fimmu.2019.02308

**Published:** 2019-09-24

**Authors:** Zhiyong Ma, Ejuan Zhang, Shicheng Gao, Yong Xiong, Mengji Lu

**Affiliations:** ^1^Department of Infectious Diseases, Zhongnan Hospital of Wuhan University, Wuhan, China; ^2^Wuhan Institute of Virology, Chinese Academy of Sciences, Wuhan, China; ^3^Institute of Virology, University Hospital Essen, University of Duisburg-Essen, Essen, Germany

**Keywords:** hepatitis B virus, functional cure, B cell response, therapeutic vaccine, chronic hepatitis B

## Abstract

The central role of the cellular immune response in the control and clearance of the hepatitis B virus (HBV) infection has been well-established. The contribution of humoral immunity, including B cell and antibody responses against HBV, has been investigated for a long time but has attracted increasing attention again in recent years. The anti-HBs antibody was first recognized as a marker of protective immunity after the acute resolution of the HBV infection (or vaccination) and is now defined as a biomarker for the functional cure of chronic hepatitis B (CHB). In this way, therapies targeting HBV-specific B cells and the induction of an anti-HBs antibody response are essential elements of a rational strategy to terminate chronic HBV infection. However, a high load of HBsAg in the blood, which has been proposed to induce antigen-specific immune tolerance, represents a major obstacle to curing CHB. Long-term antiviral treatment by nucleoside analogs, by targeting viral translation by siRNA, by inhibiting HBsAg release via nucleic acid polymers, or by neutralizing HBsAg via specific antibodies could potentially reduce the HBsAg load in CHB patients. A combined strategy including a reduction of the HBsAg load via the above treatments and the therapeutic targeting of B cells by vaccination may induce the appearance of anti-HBs antibodies and lead to a functional cure of CHB.

## Introduction

It is estimated that more than 250 million people worldwide are chronically infected with the hepatitis B virus (HBV), and over 887,000 deaths are caused by HBV infection annually due to the disease's complications, such as cirrhosis and hepatocellular carcinoma (HCC) (1, 2). Up to 95% of adults are spontaneously cleared of the virus after an acute self-limiting HBV infection, while 90% of newborns develop a chronic infection. The outcome of acute HBV infection is mainly determined by the strength and breadth of the host's adaptive immune responses against the virus (3). An acute resolving HBV infection is always associated with multi-specific and vigorous HBV-specific CD8^+^ T cell responses (4–6), which are functionally exhausted during chronic HBV infection partly due to the high load of viral antigens in the blood (7–9). While the central role of the CD8^+^ T cell response in the control of HBV infection is well-established, the contribution of humoral immunity (including B cell and antibody responses) has largely been neglected. Recently, several groups have addressed this question and highlighted that HBV-specific B cells are present in the blood and liver but functionally impaired during chronic HBV infection (10–12). Moreover, the level of the serum hepatitis B core antigen (HBcAg) antibody (anti-HBc) was to be found positively correlated with HBV-induced liver disease or inflammation (13, 14) and predicted the therapeutic efficacy of peginterferon (Peg-IFN) and nucleos(t)ide analogs (NAs) in hepatitis B e antigen (HBeAg)-positive chronic hepatitis B (CHB) patients (15–17).

Currently, Peg-IFN and NAs are approved and recommended as first-line therapies for CHB in clinical guidelines (18). However, it is difficult to cure CHB with these drugs, which means achieving sustained undetectable HBV surface antigen (HBsAg) and HBV DNA levels in serum, with or without the appearance of antibodies to the HBsAg (anti-HBs) (19). Anti-HBs antibodies were initially recognized as a diagnostic marker, indicating protective immunity after acute-resolving HBV infection or vaccination. Now, they are considered a biomarker for a functional cure of CHB. Thus, the therapeutic targeting of HBV-specific B cells and the induction of an anti-HBs antibody response are essential parts of a rational strategy to cure CHB. Knowledge of the features of B cell responses against HBV during acute and chronic viral infections is needed to rationally design strategies that target B cells in CHB patients. In this review, we summarize the currently available information about host B-cell and antibody responses in HBV infections, as well as their relationship with HBV pathogenesis, the pivotal role of HBsAg in HBV pathogenesis, and immunotherapeutic approaches to induce HBV-specific immunity and anti-HBs antibodies in chronic HBV infection.

## Does B Cell-Mediated Humoral Immunity Play a Key Role in HBV Control and Clearance?

Unlike HBV-specific CD8^+^ T cells, which can be detected by tetramer staining *ex vivo* or after *in vitro* expansion, the specific B cells that target HBV have only been studied recently by using fluorochrome-labeled HBV proteins (10–12). However, several clinical observations suggest that B cell response may have an important role in the control of HBV infection. Rituximab, an antibody of CD20, is widely used to deplete B cells during chemotherapy in B cell lymphomas or autoimmune diseases. If these patients have a previously controlled HBV infection, the clinical application of rituximab may cause HBV reactivation, indicating that B cell responses against HBV are essential to maintaining effective host immune control over HBV (20–22). Moreover, anti-HBs positivity is associated with a decreased risk of HBV reactivation in these patients, suggesting that anti-HBs may also prevent the HBV reactivation (23). Indeed, anti-HBs are known as protective antibodies, which block HBV entry into host hepatocytes and clear infectious HBV particles *in vivo* (24). Several B cell epitopes have been identified on three large, middle, and small HBV surface proteins. One well-defined region called the a-determinant comprises a number of conformation-dependent epitopes, which are located within the first loop amino acid (aa)124-137 and the second loop aa139-147 of HBsAg. The majority of anti-HBs antibodies developed by vaccination recognize the a-determinant. Another important region is located within the sequence aa21-47 of the HBV large surface protein, which contains the binding site of the HBV cellular receptor, sodium-taurocholate co-transporting polypeptide (NTCP). Thus, the antibodies specific to this region may have potent neutralization activities (24, 25). These studies imply that the B cell-mediated humoral immune response represents an important component in the sustained control of HBV infection.

However, whether the B cell-mediated immune response directly influences the HBV immunopathogenesis remains unclear. In patients with HBeAg-positive CHB, the appearance of the HBeAg antibody (anti-HBe) has been known to be an indicator of a low level of hepatic HBV replication, which is usually associated with the clinical remission of liver disease and a favorable outcome (2). Recently, the level of the hepatitis B core antigen (HBcAg) antibody (anti-HBc) in CHB patients was found to be positively correlated with HBV-induced liver disease or inflammation in CHB patients (13, 14). Consistently, the baseline anti-HBc level was found to be a useful predictor of Peg-IFN and NAs therapy efficacy in HBeAg-positive CHB patients (15–17). In a recent study, a higher baseline anti-HBc titer could predict a higher rate of spontaneous HBeAg seroconversion in HBeAg-positive children with a normal alanine aminotransferase (ALT) level. The anti-HBc level could reflect the strength of the anti-HBV immune response in the HBeAg-positive normal ALT phase of CHB (26). A study using liver samples from different phases of CHB was performed to determine the intrahepatic gene signatures. The results demonstrated that a high up-regulation of immunoglobulin-encoding genes and B-cell function-related genes was found in the immune active phase compared with the immune tolerant and inactive carrier phases (27). Moreover, a powerful B cell response with a massive accumulation of plasma cells secreting IgG and IgM to HBcAg was found in the liver of two patients with HBV-related acute liver failure (28, 29). In a very recent study, Le Bert et al. showed that the frequencies of HBcAg-specific B cells, but not HBsAg-specific B cells, were temporarily increased in the blood during hepatic flares in four CHB patients (12), again indicating a possible link between HBcAg-specific B cells and liver damage in CHB patients. These data suggest that humoral immunity may participate in the pathogenesis of HBV infection. It remains to be investigated whether the anti-HBc antibody response is, indeed, the initial trigger of the host's immune attack against HBV in the liver, thereby leading to inflammation and immunopathological processes in chronically HBV infected patients.

Given the importance of anti-HBs in preventing and curing HBV infection, several groups have attempted to detect the HBsAg-specific memory B cell response using different methods in vaccinated people and CHB patients. An HBs-ELISPOT assay has been developed to identify anti-HBs secreting B cells by culturing enriched CD19^+^ cells with stimulation of CD40-CD40L for 5 days (30, 31). Another group directly detected HBsAg-specific memory B cells by FACS analysis *ex vivo* utilizing HBsAg-conjugated microbeads in an enriched CD19^+^ cell population (32). Apparently, the frequency of HBsAg-specific memory B cells was extremely low. These two methods efficiently detected HBsAg-specific B cells in HBsAg-vaccinated individuals but not in patients with chronic HBV infection. Recently, two groups utilized fluorochrome-labeled recombinant HBsAg as “bait” to detect and characterize HBsAg-specific B cells by flow cytometric analysis *ex vivo* in HBV-infected patients (10, 11). Similar results were obtained from the two studies, demonstrating that the frequencies of HBsAg-specific B cells in the blood were similar in acute, chronic, and resolved HBV infection, and have no correlation with the serum levels of HBsAg, HBV DNA, and ALT. The phenotype of HBsAg-specific B cells from CHB patients resembled “atypical memory B cells” that are characterized by a low expression of CD21 and CD27 but a high expression of inhibitory markers, such as programmed cell death receptor-1 (PD-1) and the transcription factor, T-bet. Moreover, HBsAg-specific B cells from CHB patients were unable to mature into anti-HBs-secreting cells *in vitro*. However, their function could be partially restored by specific culture conditions, such as a PD-1 blockade or the addition of IL-2, IL-21, and CD40L-expressing feeder cells (10, 11, 33). The dysfunction and phenotype change of B cell responses during chronic HBV infection were also confirmed by other groups (34–36). Le Bert et al. conducted a more comprehensive characterization of B cell responses against HBsAg and HBcAg in CHB patients (12). They found that HBcAg-specific B cells are present at a higher frequency than HBsAg-specific B cells in CHB patients. Furthermore, nearly all HBcAg-specific B cells are the IgG+ memory B cell phenotype and mature efficiently into antibody-secreting cells *in vitro*. The differences in the phenotype and function between HBsAg- and HBcAg-specific B cells in the same patient suggest that a high level of HBsAg might cause dysfunctional programming of HBsAg-specific B cells through persistent stimulation (12). The characterization of the HBsAg-specific B cell response in chronic HBV infection facilitates the development of strategies that target B cell responses to induce anti-HBs antibodies for the functional cure of CHB.

Using highly sensitive immunoassays, previous studies demonstrated the existence of anti-HBs antibodies in CHB patients, but they are mainly detected as being complexed to HBsAg (37, 38). This result may suggest that anti-HBs antibodies are produced by HBV-specific B cells in chronic HBV infection but masked by the high level of circulating HBsAg. This may lead to the coexistence of HBsAg and anti-HBs antibodies in CHB patients when their specificity does not match, as shown by several studies (39, 40). In some cases, the appearance of anti-HBs antibodies alone may clear HBsAg in the peripheral blood, but they do not terminate chronic HBV infection in the liver (41). Thus, the HBV-specific B cell response is an integrative part of the host's defense and contributes to HBV pathogenesis, clearance, and protective immunity but is not sufficient to control HBV infection alone.

## The Efficacy of Antibody-Medicated Immunotherapy or Triggering B-Cell Response to Cure Chronic Hepatitis B: Results from Clinical Studies

Polyclonal hepatitis B immunoglobulins (HBIG) are prepared from the pooled plasma of vaccine recipients with a high titer of anti-HBs antibodies. HBIG has been widely used in clinical environments for post-exposure prophylaxis against HBV infection in neonates born to HBsAg carriers (42), as well as in HBV infected liver transplant patients (43). The success of HBIG in these conditions raises the possibility of antibody-mediated immunotherapy against chronic HBV infection (25). Indeed, three pioneering studies tested the efficacy of anti-HBs monoclonal antibodies or HBIG in CHB patients, alone or in combination with alpha-interferon (44–46). All these studies demonstrated the temporary reduction of HBsAg and HBV DNA after a high dose of anti-HBs antibody treatment, though the long-term clearance of HBsAg was not observed. Particularly, in a phase I clinical study, a mixture of two monoclonal antibodies, named HBV-AB^XTL^, was well-tolerated and led to a significant reduction of serum HBsAg and HBV-DNA after repeat administration (45). A phase II clinical study was also conducted to evaluate the therapeutic efficacy of a combination of HBV-AB^XTL^ with antiviral treatment in chronic HBV-infected patients. However, the results have not yet been released. In a recent pilot study, eight lamivudine-treated CHB patients received monthly HBIG injections followed by an HBV vaccination, which led to a significant decrease of serum HBsAg in half of the patients after 1-year of treatment (47). Importantly, three patients became anti-HBs positive, thereby achieving the goal of a functional cure (47). This study implied that a combination strategy with antiviral treatment and antibody mediated immunotherapy, followed by triggering a B cell response through vaccination may lead to a sustained loss of HBsAg and a functional cure for CHB. However, further clinical trials are needed to test and confirm the therapeutic efficacy of this combination strategy.

As mentioned above, the majority of patients with persistent HBV infections are always linked with the dysfunction of HBV-specific T-cell and B-cell responses (3). During the past two decades, repeated attempts have been made to restore efficient HBV-specific T-cell and B-cell responses in such patients, using conventional or modified HBV vaccines. These attempts have been called “therapeutic vaccination,” with the goal of stopping chronic HBV infection. Using a mammalian cell expressed recombinant Pre-S2/S protein, Pol et al. were the first to demonstrate that vaccination with this protein leads to a significant decrease, or the disappearance, of serum HBV DNA in 50% of treated patients in a pilot study (48). However, the treatment's therapeutic efficacy was not confirmed in a multicenter study including 118 CHB patients (49). Six immunizations with a vaccine consisting of recombinant HBsAg with anti-HBs immune complexes (HBsAg-IC) induced a higher rate of HBeAg seroconversion in HBeAg-positive CHB patients compared to the control group (50). However, in a larger scale phase III study, the therapeutic efficacy of HBsAg-IC was not confirmed, though the vaccination was performed at a high dose and given 12 times (51). Antiviral therapy with NAs could partially restore the HBV-specific T cell response in CHB patients (52–54), so it is rational to enhance the efficacy of therapeutic vaccination through combination with antiviral therapy.

Co-administration of the HBsAg/AS02B adjuvant candidate vaccine with lamivudine induced a vigorous HBsAg-specific T cell response in patients with HBeAg positive CHB. However, this vaccination strategy did not demonstrate superior clinical efficacy to improve the HBeAg seroconversion rate when compared to treatment with lamivudine alone (55). In another study, 180 HBeAg positive patients were randomly assigned into three groups to receive a mammalian cell-derived vaccine containing PreS1/PreS2/S proteins (named Sci-B-Vac™), lamivudine monotherapy, or a combination treatment. The PreS1/PreS2/S vaccine showed improved immunogenicity and could rapidly induce higher levels of anti-HBs antibodies than the conventional HBsAg vaccines in previous studies (56, 57). The results demonstrated that a combination treatment with the PreS1/PreS2/S vaccine and lamivudine led to enhanced efficacy in viral inhibition, although the HBeAg seroconversion rate was not different. Moreover, anti-HBs antibodies were detected in 55/120 vaccine recipients. The appearance of anti-HBs was associated with significantly higher HBeAg seroconversion rates and a greater suppression of HBV DNA levels in these patients (58). This study highlighted that anti-HBs antibodies could be induced by therapeutic vaccination and might play an important role in the suppression of viral replication.

Taken together, these various clinical trials mentioned suggest that antibody-mediated immunotherapy or targeting B cells by therapeutic vaccination may reconstitute the HBV-specific immune response and lead to a reduction of HBV replication in some CHB patients. However, the efficacy of these treatments is low, and further efforts are needed to optimize the therapeutic efficacy.

## The Pivotal Role of HBsAg in the Pathogenesis of Chronic HBV Infection and the Suppression of Effective Host Immunity

**Figure 1:**
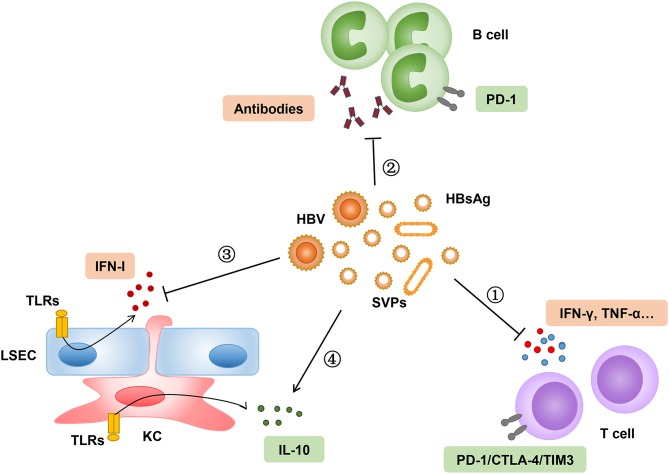
High load of HBsAg suppresses innate and adaptive immune responses through different mechanisms. (1–2) Persistent stimulation of HBsAg leads to exhaustion of HBsAg-specific T-cell and B-cell responses, which are characterized by upregulation of inhibitory molecules, such as PD-1, cytotoxic T-lymphocyte associated protein 4 (CTLA-4), and T cell immunoglobulin domain and mucin domain-containing molecule- (TIM-) 3. (3–4) High level of HBsAg attenuates hepatic innate immunity through inhibition of TLR-mediated signal pathway and induction of interleukin-10 in LSECs and KCs. SVPs, subviral particles.

Following viral entry into the hepatocytes, the HBV relaxed circular DNA (rcDNA) is converted into a covalently closed circular DNA (cccDNA) minichromosome, which is serviced as a template for subsequent transcription and translation of viral proteins. Many viral proteins were synthesized and secreted into the serum, including three HBV surface proteins and the hepatitis B core-related antigen (HBcrAg), which contains HBeAg (59). The serum level of HBcrAg has been shown to correlate with HBV cccDNA transcriptional activity in CHB patients (60, 61). Furthermore, CHB patients with a persisting high level of HBcrAg during antiviral therapy have an increased risk to develop HCC despite sustained viral suppression via long-term NAs treatment (62, 63). The clinical significance of HBsAg quantification during chronic HBV infection and antiviral treatment has been studied extensively (64–66). The HBsAg level is the highest in the immune tolerance phase and starts to decline slowly during the immune clearance phase. The HBsAg level progressively decreases after spontaneous or antiviral therapy induced HBeAg seroconversion. Accumulating evidence suggests that high levels of HBsAg may have an immunosuppressive role on both innate and adaptive immunity against HBV (67–69). Indeed, HBsAg has been shown to suppress innate hepatic immunity by inhibiting the toll-like receptor (TLR) mediated signal pathways in Kupffer cells (KCs) and sinusoidal endothelial cells (LSECs) (70, 71). The function of myeloid dendritic cells (DCs) was also impaired to stimulate T cell responses in the presence of HBsAg *in vitro* (72). However, in another study, the DCs isolated from CHB patients showed a normal ability to stimulate the expansion of autologous HBV-specific T cells through the cross presentation of circulating HBsAg (73). The available results about the HBsAg-mediated suppression of innate immunity need to be carefully interpreted, because the majority of experiments were performed *in vitro*. In general, CHB patients are immunologically intact and do not show a higher susceptibility to bacterial or other opportunistic infections. The dysfunction and exhaustion of HBV-specific CD8^+^ T cell responses is a hallmark of chronic HBV infection. The high load of circulating and hepatic HBsAg may contribute to the impairment of HBsAg-specific CD8^+^ T cell response through persistent antigen stimulation (74–76). Moreover, HBsAg may suppresses T cell responses by promoting the differentiation of monocytes into myeloid-derived suppressor cells (MDSCs) and enhance the regulatory T cell response (77, 78). In a woodchuck hepatitis virus (WHV) transgenic mouse model, the high level of virus replication and protein expression in male mice induced the expansion of intrahepatic regulated T cells, leading to the impairment of WHV-specific CD8^+^ T cell responses and gender-related differences in the outcomes of viral infection (79). As mentioned above, a high level of HBsAg might lead to the dysfunctional differentiation of HBsAg-specific B cells, but not HBcAg-specific B cells in CHB patients (12). These studies demonstrated that the high HBsAg load may induce HBsAg-specific immune tolerance through different mechanisms and may represent a main obstacle in curing CHB (Figure 1).

Several studies demonstrated that quantitative baseline HBsAg levels and their changes during the early phase of antiviral therapy could predict therapeutic efficacy, as well as the clearance of HBsAg in CHB patients (64, 65, 80–83). Moreover, lower levels of serum HBsAg at the end of antiviral therapy were associated with a higher rate of HBsAg loss in HBeAg-negative CHB patients after the cessation of long term NAs treatment (84, 85). Although NAs treatment efficiently inhibits HBV replication, it has little effect on the secretion and clearance of HBsAg (86, 87). Peg-IFN therapy may increase the rate of HBsAg loss in low level HBsAg patients, which, in previous studies, has been highlighted in both switch to or add-on strategies combining IFN and NAs to cure CHB (88–90). In patients experiencing HBsAg seroclearance, the Peg-IFN therapy or a cessation of NAs treatment enhanced the natural killer cell's functionality and increased HBV-specific T cell responsiveness (90–94). These results suggest that a sequential combinatorial therapy should ideally cause a precipitous decrease in HBsAg that, when followed by an immunomodulatory therapy, leads to sustained HBsAg loss (95).

## How to Optimize the Therapeutic Efficacy of Strategies Targeting B Cell Responses in CHB Patients

Due to the low efficacy of therapeutic vaccination targeting B cell responses in chronic HBV infection, we need to optimize the efficacy of this strategy to achieve a functional cure for CHB. Several approaches, including the reduction of circulating HBsAg, improvement of B-cell targeting vaccines, a combination with immunomodulation, and a rational selection of patients could be employed to boost the therapeutic efficacy of B cell targeting vaccination in CHB patients.

**Figure 2:**
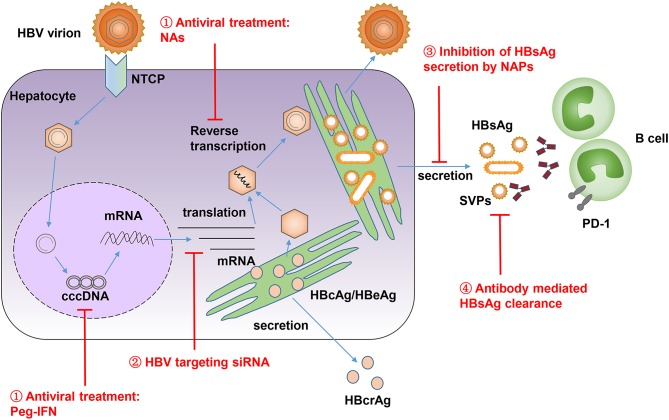
HBV life cycle is shown and different approaches are developed to reduce high level of HBsAg in CHB patients. (1) Antiviral treatment by NAs and Peg-IFN, which suppress HBV reverse transcription and target the epigenetic regulation of HBV covalently closed circular DNA (cccDNA). (2) Suppression of HBsAg expression by HBV-targeting siRNA. (3) Inhibition of HBsAg release by NAPs. (4) Antibody mediated reduction and clearance of HBsAg. SVPs, subviral particles; NTCP, sodium-taurocholate co-transporting polypeptide; HBcrAg, hepatitis B core-related antigen.

Currently, long-term NAs treatment in CHB patients can lead to a gradual decrease in the serum level of HBsAg. However, only a few patients can reach a significant decrease in serum HBsAg (86, 87). Thus, we need more potent strategies to reduce the serum level of HBsAg. Several approaches were developed to allow a temporary reduction of circulating HBsAg, including targeting viral translation via siRNA, inhibiting HBsAg release via nucleic acid polymers (NAPs), or neutralizing HBsAg with specific antibodies (Figure 2) (68). Recently, an RNA interference based therapeutic agent ARC-520 has been developed. ARC-520 shows a strong efficacy in reducing HBsAg levels in treatment-naïve CHB patients who are positive for HBeAg. However, their therapeutic efficacy is compromised in patients who are HBeAg-negative or have received long-term therapy with NAs (96, 97). Moreover, the clinical development of ARC-520 was put on hold by the U.S. Food and Drug Administration (FDA) due to the delivery vehicle EX1, which probably led to the deaths of non-human primates in another study (98). NAPs are single stranded phosphorothioate oligonucleotides that can block viral entry in many viruses, including the hepatitis B and hepatitis D virus (HDV), and also have the unique ability to inhibit the release of HBsAg from HBV-infected hepatocytes (99, 100). One NAP named REP 2139 was selected and evaluated in clinical studies for its therapeutic efficacy in CHB patients (101, 102). In an open label, non-randomized study, weekly intravenous administration of REP 2139 led to a dramatic decrease of serum HBsAg and HBV DNA in 12 HBeAg-positive CHB patients, accompanied by HBsAg seroconversion (101). In another study with 12 HBV and HDV co-infection patients, the combination of REP 2139 and peg-IFN led to a reduction of HBsAg by 2–7 logs and undetectable HDV RNA in 11 patients during therapy. One year after treatment, five patients maintained negative HBsAg, five patients had high titer anti-HBs, and seven patients remained HDV RNA negative (102). These results suggest that REP 2139 has a strong ability to decrease circulating HBsAg. However, the treatment's efficacy and side effects should be further evaluated in larger scale, blind, and randomized or real-world studies. The efficacy of specific antibody mediated reduction of HBsAg has been described in a previous section of this review. It is shown that using HBIG or monoclonal antibodies against HBsAg can efficiently decrease the serum level of HBsAg in CHB patients (44–47). In addition, high affinity human anti-HBs and anti-Pre-S1-monoclonal antibodies have been developed recently, and these antibodies have shown an excellent ability to efficiently clear circulating HBsAg in different mouse models (103–106). These studies emphasize the new interest in developing neutralizing antibodies as a therapeutic strategy to reduce circulating HBsAg.

Besides the reduction of circulating HBsAg to overcome immune tolerance in CHB patients, the improvement of B-cell targeting vaccines is the second step to optimize the therapeutic efficacy of this combination strategy. Therapeutic vaccination with conventional yeast derived HBsAg vaccines showed limited efficacy for HBsAg seroclearance in CHB patients, even when combined with antiviral treatment (55, 107). New generation HBV vaccines containing one (PreS2) or two (PreS1 or PreS2) additional envelope proteins have been developed in transfected mammalian cells. Compared with conventional vaccines, new generation vaccines display more immunogenic properties and rapidly induce higher levels of anti-HBs antibodies both in healthy individuals and in non-responders to yeast-derived vaccines (49, 57, 108). As described in the previous section of this review, the PreS2 containing HBV vaccine had limited therapeutic efficacy in HBeAg positive CHB patients in the absence of antiviral treatment (49, 109, 110). In another study, a PreS2-S vaccine combined with IFN-alpha-2b showed a better potential benefit in children with CHB than IFN-alpha-2b monotherapy (111). In contrast, the PreS1-PreS2-S vaccine Sci-B-Vac™induced anti-HBs antibodies in nearly 50% of vaccine recipients in HBeAg positive CHB patients, even though these antibodies had a poor ability to neutralize the circulating HBsAg (58). However, the appearance of anti-HBs was associated with significantly higher HBeAg seroconversion rates and a greater suppression of HBV DNA levels in these patients (58). A nasal vaccine candidate (NASVAC), comprising HBsAg and HBcAg, has been shown to be safe and highly immunogenic in healthy volunteers (112). The administration of NASVAC through intranasal and subcutaneous routes led to sustained negative HBV DNA in 50% patients with CHB (113). In a phase III clinical study, the therapeutic efficacy of NASVAC was comparable to Peg-IFN in reducing HBV DNA under the limits of detection (114). It is necessary to further confirm the therapeutic efficacy of NASVAC on a large scale and in multicenter studies. Current studies have demonstrated the importance of improving the therapeutic efficacy of B-cell targeting vaccines.

The crosstalk of TLR7/9 and the B cell receptor (BCR) signal pathway in B cells has been previously described; this crosstalk may modulate the B cell's response to foreign or endogenous antigens (115, 116). The stimulation of B cells with a TLR7 ligand and antigen induced a more robust increase in germinal center B cells, plasmablasts, plasma cells, and serum antibodies, compared to their cohorts who received antigen alone (117). A similar intrinsic signal pathway via TLR9 in B cells was also described. This pathway increased production of IgG, in particular, with a shift to the IgG2a subclass (118, 119). These results support the use of TLR7/9 ligands as adjuvants to enhance the therapeutic efficacy of B-cell targeting vaccination (120). Indeed, the immunogenicity of HBsAg was enhanced by a mixture of TLR9 agonists CPG 7909 and 1018 ISS. New vaccines induced a higher titer of anti-HBs accompanied by increased avidity through modulation of the late affinity maturation process (121–124). Importantly, this new vaccine formula proved more effective in immune-suppressed populations (125–127). Further studies are needed to explore the therapeutic efficacy of these improved vaccines on CHB patients.

The lessons from clinical studies suggest that patients with low levels of HBsAg easily reach the goal of HBsAg loss after ceasing long-term NAs treatment (84, 85) or switching to Peg-IFN therapy (82, 83). Thus, the rational selection of patients with low levels of serum HBsAg after long term NAs treatment could be employed to optimize the therapeutic efficacy of B cell targeting vaccination. Indeed, in a recent study, conventional HBsAg-based vaccination in 20 HBeAg-negative patients with HBsAg <1,000 IU/ml, resulted in a significant HBsAg decline in 14 patients and HBsAg loss in 2 patients (128). In another study, the switch from long-term entecavir (ETV) treatment to a combined therapy with IFN-alpha-2b, HBsAg-based vaccination, and IL-2 resulted in a higher HBsAg loss rate (9.38%) compared to IFN-alpha-2b (3.03%) alone or continued entecavir (3.70%) therapy in HBeAg-negative patients. Moreover, among patients with baseline HBsAg titers ranging from 100 to 1,500 IU/mL, the HBsAg loss rate was 27.3% in the combination therapy group (90). These studies suggest that in long-term NAs treated CHB patients, particularly those with low baseline HBsAg levels, therapeutic vaccination with conventional HBsAg may enhance HBsAg loss. In a recent study, CHB patients under NAs treatment (and with low HBsAg levels) were selected for a pilot immunization study with Sci-B-Vac™ (129). Three vaccinated patients developed anti-HBs and were cleared of HBsAg after the vaccination. Though this study only involved a few patients, the result is encouraging and hints at the potential usefulness of this approach.

## Conclusions

HBV affects more than 250 million people worldwide and represents a major global public health concern. Current antiviral therapies with Peg-IFN and NAs can suppress HBV replication and improve the prognosis of CHB, but they can hardly clear HBsAg to achieve a functional cure of CHB. During chronic HBV infection, HBV-specific T-cell and B-cell responses are functionally impaired, which leads to a limited efficacy of B-cell targeting therapeutic vaccination in CHB patients. A high level of circulating and hepatic HBsAg may contribute to HBV-specific immune tolerance and represents a main obstacle in curing CHB. Declining HBsAg levels were found to be associated with a higher chance to achieve a sustained response in CHB patients under IFN treatment (130). Moreover, a lower level of serum HBsAg at the end of the NAs treatment was associated with a higher rate of HBsAg loss in HBeAg-negative CHB patients after cessation of NAs treatment or a switch to Peg-IFN therapy. The reduced HBsAg level in these patients is attributed to the effective removal of HBV cccDNA in patients and considered to be a useful biomarker. At the same time, a reduced serum HBsAg level may also facilitate the recovery of the host's immune system and help control HBV. Thus, the reduction of HBsAg by siRNA, NAPs, antibody-mediated neutralization, or long-term NAs treatment may overcome HBsAg-specific immune tolerance and optimize the therapeutic efficacy of B-cell targeting vaccination in CHB patients. Therefore, a sequential combination therapy strategy with antiviral treatment, a reduction of HBsAg, and therapeutic vaccination against envelope proteins may induce the appearance of anti-HBs antibodies and lead to a functional cure for CHB.

### Author Contributions

ZM, EZ, and ML designed and wrote the paper. EZ designed and drew the figure. ML, SG, and YX carefully revised the paper.

### Conflict of Interest

The authors declare that the research was conducted in the absence of any commercial or financial relationships that could be construed as a potential conflict of interest.
